# Observations on the Schirrous-Contracted Rectum

**Published:** 1809-10

**Authors:** W. White

**Affiliations:** Bath


### *Observations on the Schirrous-contracted Rectum; by Mr.* White.

I AM persuaded there is no humane practitioner who hath seen this disease, but must feel desirous of contributing his aid towards extending the knowledge of so distressing a malady; especially, as an early discovery of it may be the means of saving many of our fellow-creatures from extreme sufferings, and the fatal consequences attendant on so terrible a complaint.

It may be, perhaps, a too generally received opinion that this disorder seldom occurs, because it has been so little noticed in modern systems of Surgery. I am, however, convinced from experience and observation, that the principal reason why it is not more generally known, is owing to an imperfect investigation, in consequence of which, it is mistaken for other complaints of the alimentary canal, that are attended with similar symptoms. Out of upwards of thirty-six thousand patients, who have come under my immediate notice in the course of twenty-three years, I have met with only ten cases of Schirrous and contracted Rectum: but then it should be observed at the same time, that all of these cases have occurred within the last five years, from which circumstance, it may be fairly inferred that the disease is frequently overlooked.

Practitioners unacquainted with the disorder, will be apt to attribute symptoms of its early stage to habitual costiveness, or hæmorrhoidal affections, and those attendant on its more advanced progress to chronic diarrhœa, or dysentry. Van Swieten remarks, "It is even highly credible that such schirrous swellings lurk oftener within the bowels than is commonly believed, and there give birth to the most stubborn maladies." It is evident from cases which are recorded, that some of the old practical writers were acquainted with this disease, though they have not given us a complete history of it, as may be seen in Wiseman's Chirurgical Work, and in Van Swieten's Commentaries.

We are, however, greatly indebted to Mr. Sherwen for a very valuable paper written by him, and published in the second volume of the Memoirs of the London Medical Society, wherein the symptoms of this disorder towards its ultimate progress, are detailed with much accuracy and minuteness.

It appears from what I have seen, that persons of different temperaments, age, and sex, are indiscriminately attacked with this complaint, though most commonly those in the decline of life: and as Mr. Sherwen observes, "it comes on in the most gradual and imperceptible manner; slow in its progress, but terrible in its consequences, it yields not to medical assistance, but must, under the best management, become ultimately fatal. It admits however of palliation, and if early discovered, will also admit of the last moments of the patient being rescued from unavailing, mistaken, and distressing attempts to cure. It is therefore an object worthy of the most serious attention of every humane practitioner."

"There is no disease to which the human frame is incident that is more liable to be misunderstood. Diarrhœa, dysentery, tenesmus, cholic, painful distention of the abdomen, inflammation in the bowels, and iliac passion, which are each of them formidable, and often fatal diseases in themselves, may be successive symptoms only of the schirrous rectum. Under some one of these appearances it is highly presumable, that many patients have died without the real cause having ever been assigned or suspected; and even when it is suspected, and becomes an object of manual investigation, it may easily be mistaken for an enlargement of the prostate gland, or a schirrous uterus.

"The patient gradually experiences a difficulty in evacuating fæces of a thin consistence. There is a principle of accommodation in the human system which enables him to go on for a great length of time without applying for aid. As the passage becomes obstructed, the fæces acquire a thinner consistence, and the first complaint he makes, is of a looseness."

I shall here take the liberty of remarking on the preceding passage, that although it is true this disorder sometimes arrives at the above mentioned stage, before application is made for relief, yet it does not follow from thence, that a diarrhœa is the primary symptom: because the history of cases clearly demonstrates, that the complaint does exist for a considerable length of time before a diarrhœa comes on.

The symptoms which indicate the presence of this disease in its early stage, and which may be deemed pathognomonic, are habitual costiveness, pain in the bowels, particularly in the course of the transverse arch of the colon, and sigmoid flexure; flatulency; uneasiness in the rectum, rectum, attended with some difficulty in expelling the fæces, which gradually become scanty, smaller figured than naturally, and often discharged with a squirt.

In the fourth volume of the Memoirs of the Society, there are likewise some observations worthy of notice, by Mr. Robert White. After enumerating the principal symptoms of the disease as described by Mr. Sherwen, he observes, " But, from what I have learnt in this disorder, when these symptoms supervene, particularly that of scanty alvine excretion, it admits only of temporary relief. The point therefore to be aimed at in this complaint, is to ascertain a train of symptoms which collectively may lead to an early detection. I am also of opinion that the following symptoms will urge the necessity of manual investigation, by inducing a suspicion of the malady: When a person somewhat advanced in life is troubled with frequent constipation, complains of fulness and weight in the stomach, with repeated inclination to discharge the contents, and uneasy rumbling in the belly, and distention in the lower part of it, with a sensation of numbness towards the upper part of the sacrum, extending down the rectum, repeated fruitless efforts being also made to pass a stool, attended with a sense of constriction and tenesmus, high up in the rectum, and the flatus which seemed to the patient to occupy the intermediate space, bursts forth; clysters failing as well as medicines, and the complaint unattended with fever or pain; it will be reasonable to expcct some mechanical obstruction in the passage. This opinion will be farther strengthened, if the above symptoms return without evident cause, no well formed stool regularly intervening."

I shall now resume the history, as further detailed by Mr. Sherwen. " He continues in other respects in good health; his appetite is but little impaired; reiterated scanty evacuations, amounting in the whole to a sufficient quantity to keep the stomach easy, preserve a sort of balance in the intestinal canal; but by degrees the cavity of the gut becomes less permeable; opiates and testaceous powders have, perhaps, been had recourse to, and the frequent needing to stool abates. The patient and his friends flatter themselves that he is getting well, but he now gradually falls off in his appetite for food. The absence of stools is for some time attributed to this cause, till the lower part of the abdomen, by degrees, acquires a remarkable prominency, attended with uncommon rumbling of wind in the belly, like the

 gurgling gurgling of water in a bottle.* These two last circumstances perhaps afford pathognomonic symptoms of this disease, more especially when accompanied with frequent but scanty discharges of thin, dark coloured, slimy fæces; often not more than a tea-spoonful, seldom exceeding at one discharge a larger quantity than a table-spoonful. By degrees a total suppression of stools takes place; the tumour in the abdomen increases; the uncommon rumbling of wind becomes more audible, so as to egagage the attention of the friends and visitants of the patient. The distention gradually increases, till the stomach is oppressed, and a vomiting comes on. The vomiting is not very frequent at first; but by degrees, every thing swallowed is vomited up. Severe pains are felt from distention in various parts of the abdomen, and a true iliac passion of the chronic kind comes on, and continues as long as the patient lives, unless he is accidentally relieved by a free discharge of thin fæces, which will sometimes give respite to his sufferings; in consequence of which, the appetite for food will again return; the patient will appear getting well; but the anxious solicitude of his friends at this period, will urge him to get down a considerable quantity of generous nourishment, till a repetition of the same scene will take place, and the unhappy man is alternately tantalized and worn out either by a stoppage or a purging."

The following is particularly worthy of attention, because it will assist in discriminating this disease from a common dysentery. "A common tenesmus is generally sudden in its attack, or it follows severe purgings and dysenteries, where the preceding circumstances have been well defined. It is often the consequence of drastic cathartics, and is always attended with considerable pain, and most frequently with a mucus discharge tinged with blood, instead of fæces; whereas, that which accompanies the schirrous rectum, is attended with little or no pain, but with powerful ineffectual strainings; during which, there will often be a discharge of wind, and the mucus squeezed out is slimy, but always more or less black and excrementitious, very seldom tinged with blood. In the common tenesmus, the impetus seems entirely spent on

* This symptom has not been conspicuous in any case I have met with, and Mr. Robert White, in the paper alluded to, observes in the history of a case, that the gurgling, rumbling noise, considered by Mr. Sherwen as particular marks of this disease, were so trifling as not to be noticed, until a total obstruction took place.

on the sphincter ani, and there is more or less of a protrusion of the gut; but in a straining from schirrous rectum, the patient is not sensible of that extreme distress at the fundiment which is experienced in the other; and as soon as a small portion of excrementitious mucus is voided, he is able to rise immediately from the stool; but in a common tenesmus he is under the necessity of straining long, even after the expulsion of all that he knows from his feelings will at that effort be evacuated; and after he is able to rise from the stool, there still continues a burning pungent sensation, urging to a continual expulsion; whereas, in the tenesmus of which I am treating, after the patient has strained hard, whenever a small quantity of thin fæces arrives at the anus, it is squirted out with slight efforts, and little or no uneasiness follows; nor does the countenance shew that extreme distress attendant on a spasmodic stricture of a common tenesmus."*

If due attention be paid to the preceding description, I am persuaded the disease may be often detected in its early stage by manual investigation. On examination, different appearances will be manifested, according to the progress of the disease. In the beginning of the complaint, perhaps only a diminution of the natural diameter of the intestine will be discovered; and, although the patient may occasionally experience much pain in the bowels, attended with some difficulty in the expulsion of the fæces; yet, at this period, the fæces will not be found lessened in their diameter, because the muscular coat of the intestine is still capable of being dilated to a proper extent. Sometimes there are strictures resembling muscular rings (as in Case vi.), without any other thickening or induration of the intestine, which is ascertained on the introduction of a bougie, for the muscular action of the gut will be plainly discerned when the bougie passes thro' the strictured part: which is not the case when the rectum is thickened or indurated to any great extent, because the natural action is then lost.

Most frequently however, and unfortunately, the disease is not discovered until a very considerable thickening and induration of the intestine has taken place, which is generally in the middle and superior extremity of the rectum, where the colon terminates. Sometimes the

X 3 schirrus

* Although I have selected the most material parts of Mr. Sherwen's valuable paper relative to the symptoms of this disease, yet the whole of it deserves insertion.

schirrus fills up nearly the whole cavity of the rectum, which feels hard and uneven; and often there is an erosion of its internal membrane, with a serous or sanious discharge. I am sorry that I have not had an opportunity of inspecting the diseased parts after death; this defect, however, is amply supplied by the accurate description given by Dr. Baillie, in his Morbid Anatomy, of the general appearance of this disease on dissection. "The schirrus sometimes extends over a considerable length of the gut, viz. several inches; but generally it is more circumscribed. It exhibits the same appearances of structure which were described when speaking of the schirrus of the stomach. The peritoneal, muscular, and internal coats are much thicker and harder than in a natural state. The muscular too is subdivided by membranous septa; and the internal coat is sometimes formed into hard irregular folds. It often happens that the surface of the inner membrane is ulcerated, producing cancer. Every vestige of the natural structure is occasionally lost, and the gut appears of a gristly substance. When schirrus affects the gut, the passage at that part is always narrowed, and sometimes so much so, as to be entirely obstructed. The obliteration, or stricture, would sometimes appear to be greater than in proportion to the thickness of the sides of the diseased gut; this, most probably, depends upon the contraction of the muscular fibres of the gut, which, although diseased, have not altogether lost their natural action. When the passage is very much obstructed, the gut is much enlarged immediately above the obstruction, from the accumulation of the contents in that part of the intestine. While this disease is going on in a portion of the intestine, adhesions are formed between it and the neighbouring viscera, and the ulceration sometimes spreads from one to the other."* It appears highly probable, that the glandular structure of the rectum may form the predisposing cause of this disease; and it is also presumable, that the accumulation of hardened fæces, and their pressure on passing through the intestine, may prove the general exciting cause. Dr. Baillie observes, "There is certainly more of glandular structure in the inner membrane of the great intestines towards its lower extremity, than in any other part of it, and this sort of structure has a greater tendency

* Drawings of this disease, and appearances on dissection, may be seen in the 2d and 4th volumes of the Memoirs of the London Medical Society; and also in Mr. Bell's 1st volume of Operative Surgery.

dency to be affected with schirrus, than the ordinary structures of the body; the gut, too, is narrower at the sigmoid flexure than any other part, and therefore will be more liable to be injured by the passage of hard bodies; these, by their irritation, may excite the disease of schirrus in a part which was predisposed to it."

If the disease should be happily discovered in its early stage, and not seated beyond the lower extremity of the sigmoid flexure of the colon, much benefit may be expected from the judicious management of the surgeon: The principal indication of cure in this complaint, consists in the dilatation of the passage: hence, different methods have been proposed for the accomplishing of that end. The first case recorded by Wiseman, he attempted the dilatation of the gut with tents made of gentian root, and also of deer's suet; but these means failing, he informs us that he divided the contracted part several times with an instrument; after which the excrements came away big, and the patient was not only able to expel them well, but also to retain them; and on a subsequent examination he could not find any remains of the disease. In another case, he made use of the actual cautery, with a view to destroy a cancerous excrescence in the rectum; but afterwards the patient was seized with a pleurisy, succeeded by a dysentery, which terminated fatally.

Mr. Sherwen recommends bougies of horn, softened by means of boiling water. Dr. Darwin has recommended introducing a leathern canula in the gut, and then either a wooden maundrill, or blow it up with air so as to distend the contracted part as much as the patient can bear, or bougies made with mercurial plaster, spread on leather. Dr. Darwin has also suggested the trial of lunar caustic, as used in strictures of the urethra. Mr. Charles Bell recommends bougies made of sponge, soaked in strong mucilage, or dipped in wax; which may probably answer very well when the disease happens to be seated very low in the rectum. Mr. Bell also advises small doses of calomel to be given occasionally, and purged off once or twice a week.

Mr. Robert White likewise suggests the probability of mercury being useful in this complaint. "From analogy (he says) it may be conceived that as the employment of mercury causing a ptyalism of some duration, is so serviceable in the schirro-contracted œsophagus, benefit may be also obtained from it in the like manner in the schirro-contracted rectum."

In attempting the dilatation of the passage, I have not made use of any other means than the common rectum bougies; which I think are best calculated to answer that purpose. The young practitioner, however, must be careful to adapt the size of the bougie to the degree of obstruction in the passage, which, as far as is practicable, must be previously ascertained by introducing the finger into the rectum, and afterwards a large common bougie as high as the colon; for unless this circumstance is particularly attended to, considerable mischief may be done to the diseased part by inducing inflammation, and increasing pain. If the bougie be too large, it should be gently, slowly, but at the same time steadily introduced, until it passes the obstructed part, and enters the colon. I generally suffer it to remain about an hour, and repeat it every second or third day; for if introduced oftener, there will be danger of exciting too much irritation. If the bougie is previously warmed a little, it will yield better to the curvature of the pelvis, and pass much easier to the patient, and with less trouble to the practitioner.

It is proper to remark, that laxative medicines are not only necessary to be administered in the constipated state of the bowels attendant on the first stage of this disease, but also in its more advanced progress, when diarrhœa supervenes; because the evacuations are seldom of sufficient quantity to relieve the bowels without the aid of them. Castor oil I think preferable to any other. Aloetic purgatives should be carefully avoided, for it appears that aloes may have some share in producing the complaint, as so much of it is taken by every description of persons, and its peculiar effect on the rectum is well known.

It is also of importance to attend to the regulation of the patient's diet; and should consist (as Mr. Sherwen remarks) of that sort of food "which contains the greatest quantity of nourishment in the smallest compass." Jellies, sago, arrow root with milk, gruel with milk, beef tea, thin chocolate, fresh fish; very little animal food, fruit, or vegetables should be allowed. For common drink, water, or barley water. Every thing seasoned or salted, spirituous and fermented liquors, must be carefully avoided. The quantity of food should also be attended to, (as well as the quality) which ought to be as moderate as possible, for a strict attention in these respects will greatly tend to alleviate the sufferings of the patient.

Might not an aqueous diet be worth a trial in this disease, as as recommended by that sensible and judicious surgeon, Mr. Pearson, in cancer uteri?

CASE I. About five years since, I was requested to visit Mr. C———, about 45 years of age, who had been affected for a considerable time with pain in the rectum on going to stool, and difficulty in expelling the fæces, attended with a falling down of the gut. On examination, I found a partial prolapsus of the anus on one side, accompanied with inflammation; there was also a pouching of the integuments, as noticed by Mr. Hey in his Practical Observations on Surgery. At that time, I confess I was not experimentally acquainted with the diagnosis of schirrous rectum*, therefore did not examine farther than the prolapsed part. Means were prescribed, with a view to lessen the local inflammation; and laxatives to keep the bowels open. As the symptoms in a short time became more violent and alarming, in consequence of the increasing difficulty to the passing the fæces through the rectum, which brought on considerable distension of the abdomen, with increase of pain, and other symptoms characteristic of iliac passion, I was led to investigate more particularly the state of the rectum, which was very much indurated and contracted, as not to allow of the smallest rectum bougie to pass up farther than an inch. In the course of a few days the patient died.

CASE II. Mr. C———, about 30 years of age, applied to me about three years since, for a complaint which he supposed was the piles, that being the opinion of the medical person to whom he had previously made application; but, instead of obtaining relief, he was growing worse. He informed me, that for several years he had been of a costive habit, and had experienced some difficulty in expelling the fæces; but as that only produced a temporary inconvenience, he paid no attention; particularly, as he otherwise enjoyed perfect health. He had also observed for several years, that his stools were smaller in diameter than they had been formerly; and lately they had been discharged with a sudden squirt. His pain had now become considerable in the rectum, not only at the time of his having a motion, but at other times in the day. The former case had made so deep an impression on my mind, that I was convinced this person laboured under a schirrous rectum, particularly as soon as I saw the figure of his stools,

* Neither had I seen Mr. Sherwen's paper on this subject.

stools, which were small in diameter and scanty in quantity. On examining the rectum, I found it contracted about three inches up, and also at its superior extremity. At first I could only pass a large common bougie, but by degrees I was enabled to introduce a moderate size rectum one. For some time, he appeared very much relieved, and his stools became more copious and free, and of a natural figured appearance; and was able for a while to follow his business. Yet, however, soon afterwards, the intestine became more thickened and indurated, with increase of pain; accompanied by a thin acrid discharge, and loose stools, which sometimes passed away involuntarily. Suppuration also took place on one side of the anus, which broke externally. I advised ung. hydrag. to be rubbed on the verge of the anus. Various anodyne injections were occasionally thrown up. Cicuta, &c. were given; but nothing gave him any ease but large and repeated doses of opium. As he grew worse he was advised by his friends to go to a neighbouring infirmary. The surgeon under whose care he was admitted, treated it as a common fistula, and laid the sinus open, which, however, afforded him no relief. As soon as he was able he returned home, and sent for me, when I found his complaint greatly aggravated; and after several months severe sufferings, he died.

Case III. William Lovell, about sixty years of age, applied to me (about the same time as the last mentioned person) who had laboured under the complaint for some time, in consequence of which he was very much weakened. He was costive, and complained of great pain on his going to stool. On examination, I found the rectum in a schirrous state, and particularly contracted about the middle of the gut, and at its superior extremity. At first I could only pass a large common bougie, but by degrees a moderate size rectum one. For some time he appeared better, the fæces passing easier through the rectum. The relief, however, was not permanent, for the indurated and contracted state of the intestine increased, with increase of pain, which he laboured under several months, and then died. A short time previous to his death, a suppuration took place on one side of the rectum, and broke externally at the anus.

Case IV. I was requested about two years since, to visit Miss C———, aged 23, who complained of having frequently acute pain in the bowels, and was of so costive a habit, that she often went a week without having a motion, the expulsion of which gave her always considerable pain, pain, whether she went so long or not. Her appetite for food was very indifferent; she was often sick, and brought up bile. Not being relieved by the use of laxative medicines, I suspected the cause of the complaint to be in the rectum. On examination, I found the whole length of that intestine considerably lessened in its diameter, so much so, that it was with some difficulty that the smallest rectum bougie was made to pass; there did not however appear any sensible thickening or induration of the intestine. Bougies were occasionally introduced for some time, from which she derived considerable benefit, as her motions passed much easier and freer. About four months since I was requested to visit her again, when I found her labouring under symptoms of diseased liver, to which supervened symptoms of hydrocephalus internus, and about a week after she died.

Case V. Two years ago I was requested to visit Mrs. F———, about 35 years of age, who complained of excruciating pain in the rectum, attended with a diarrhœa, and a considerable discharge of matter. She was very much reduced, and exceedingly weak; her pulse was quick and feeble, with other symptoms of hectic fever. On examination I found the gut in a very diseased ulcerated state, and an opening in it, which communicated with the vagina. She lived a few weeks after, and her sufferings were mitigated as much as possible by the use of opium.

Case VI. Mr. C———, about 28 years of age, applied in the autumn of 1808, and informed me that he had been occasionally unwell about six years, in the course of which time he had consulted different medical gentlemen, and had taken a variety of medicines, without receiving the least benefit. He complained of pain, which was sometimes very acute, in the course of the transverse arch of the colon, and sigmoid flexure. His bowels were costive, and whenever he had a stool, it was attended with pain in the rectum, and always felt afterwards as if some fæces remained behind, which he had not been able to expel. The motions were small figured, scanty, and discharged with a squirt. His appetite was very good, but felt himself generally worse after eating a hearty meal. His strength was not sensibly diminished, as he was able to follow his business as usual. On investigation, I found the rectum considerably lessened in its diameter, as it was with some difficulty I could pass the smallest rectum bougie. There were two strictures, one about three inches up, and the other at the superior extremity of the intestine. The gut

The gut however had not an indurated feel, though it appeared lessened in its diameter. I explained to the patient the nature of the disease, and enforced the necessity of using bougies, with a view to overcome the obstructions, to which he very readily consented, particularly as every other means had failed in procuring him relief. From persevering some time in the use of the bougies, the passage was so much dilated as to admit of the introduction of the largest size; and the natural action of the intestine was so far recovered, that he often had evacuations, (without the need of taking medicines) which were more copious and easier in passing. Yet he still had occasionally returns of pain in the course of the colon, which no doubt had been weakened and distended by the long continuance of the complaint. He went into the country about three months ago, when I requested him to continue the occasional use of the bougies.

Case VII. Mr. H———, about 60 years of age, applied to me in June 1808, and complained that he had been troubled with a purging for several months, attended with considerable pain in the rectum, particularly when the fæces passed. He was very much reduced, countenance pale, and face rather œdematous. His appetite for food however remained tolerably good. By the advice of an eminent physician he had taken medicines (such as are commonly prescribed in diarrhœa) for a long time without experiencing any benefit. On examination, I found the cavity of the rectum nearly obliterated by the schirrous state of that intestine, and was only able to pass a moderate size common bougie, which I introduced a few times. The fæces often passed involuntarily, and there was also frequently a considerable sanious discharge. His pain was very great, which could only be mitigated by the use of opium. He lived about six months after I first saw him.

Case VIII. Ann Davy, about 45 years of age, complained of great pain about the anus, particularly on going to stool. Her motions were loose, and there was also a considerable serous discharge. She had been ill several months, and was daily growing worse. On investigation, the anus projected, which had a schirrous feel, and the whole length of the rectum so indurated and contracted, as scarcely to admit the introduction of a common bougie. Bougies were used a few times; but as the pain and irritability of the part increased, they were not continued. Opiates were given occasionally; and when the evacuations evacuations were not sufficient to relieve the bowels, elect. sennæ, &c. I have not heard of her for several months, as she went to live at some distance.

Case IX. Occurred about a year since; I only saw the patient three or four times. He complained of having frequently considerable pain in the course of the transverse arch of the colon, and difficulty in expelling the fæces. He was costive. Previous to my seeing him, he had been using bougies, as the disease had been discovered while he happened to be in London. The strictured parts were about the middle and superior extremity of the rectum. I advised him to continue the use of the bougies; which he was able to introduce very well himself. I have not seen him for several months.

The Tenth Case is at present under my care, and may take some future opportunity of communicating the result. The pain is considerable at the sigmoid flexure of the colon, but as yet, the rectum is not particularly indurated.

I shall conclude with remarking, that symptoms of iliac passion did not supervene to any of the cases which proved fatal, except the first.

I am, &c.

W. WHITE.

*Bath, August 29, 1809.*

