# Erratum

**Published:** 1809-11

**Authors:** 


### ERRATUM.

Page 288, line 11, place the period after,—*if the bougie be too large.*

